# A Developmental Approach to Internal Medicine Residency Education: Lessons Learned from the Design and Implementation of a Novel Longitudinal Coaching Program

**DOI:** 10.1080/10872981.2019.1591256

**Published:** 2019-03-29

**Authors:** Jed D. Gonzalo, Daniel R. Wolpaw, Karen L. Krok, Michael P. Pfeiffer, Jennifer S. McCall-Hosenfeld

**Affiliations:** a Medicine and Public Health Sciences and Health Systems Education, Penn State College of Medicine, Hershey, PA, USA; b Medicine and Humanities, Penn State College of Medicine, Hershey, PA, USA; c Medicine, Penn State College of Medicine, Hershey, PA, USA; d Medicine, Penn State Hershey Heart and Vascular Institute, Penn State College of Medicine, Hershey, PA, USA; e Medicine and Public Health Sciences, Penn State College of Medicine, Hershey, PA, USA

**Keywords:** Competency-based education, coaching, entrustment, graduate medical education, professional development

## Abstract

**Background**: Resident physicians’ achievement of professional competencies requires reflective practice skills and faculty coaching. Graduate medical education programs, however, struggle to operationalize these activities.

**Objective**: To (1) describe the process and strategies for implementing an Internal Medicine (IM) resident coaching program that evolved in response to challenges, (2) characterize residents’ professional learning plans (PLPs) and their alignment with EPAs, and, (3) examine key lessons learned.

**Design**: The program began in 2013 and involved all postgraduate years (PGY) residents (n = 60, 100%), and 20 faculty coaches who were all IM trained and practicing in an IM-related specialty. One coach was linked with 3–4 residents for three years. Through 1:1 meetings, resident-coach pairs identified professional challenges (‘disorienting dilemmas’ or ‘worst days’), reviewed successes (‘best days’), and co-created professional learning plans. Typed summaries were requested following meetings. Coaches met monthly for professional development and to discuss program challenges/successes, which informed programmatic improvements; additionally, a survey was distributed after three program years. Data were analyzed using quantitative and qualitative methodologies.

**Results**: Disorienting dilemmas and professional learning plans mapped to all 16 EPAs and four additional themes: work–life balance, career planning, teaching skills, and research/scholarship. The most-frequently mapped topics included: PGY1 – leading and working within interprofessional care teams (EPA 10), research and scholarship, and work–life balance; PGY2 – improving quality of care (EPA 13), demonstrating personal habits of lifelong learning (EPA15), and research and scholarship; PGY3 – lifelong learning (EPA15); career planning was common across all years.

**Conclusions**: Lessons learned included challenges in coordination of observations, identifying disorienting dilemmas, and creating a shared mental model between residents, faculty, and program leadership. The coaching program resulted in professional learning plans aligned with IM EPAs, in addition to other professional development topics. Operationalization of aspects of these results can inform the development of similar programs in residency education.

## Introduction

Nearly two decades ago, the Accreditation Council for Graduate Medical Education implemented the Competency-Based Medical Education (CBME) Outcomes Project to better prepare physicians-in-training for practice by focusing on observable activities rather than a less granular, ‘time-based’ framework [1–3]. In contrast to conventional approaches relying heavily on knowledge-based assessments, competencies and Entrustable Professional Activities (EPAs) allow for focused and meaningful assessment targets [4,5]. To achieve CBME outcomes, residents must develop deliberate reflective practice skills, which are necessary for critical thinking, professionalism, and continuous professional development [5–8].

Critical factors that enable the assessment of whether a learner can be entrusted include direct observation, feedback, and reflective practice[9]. Reflective practice includes thinking about and critically analyzing one’s actions with the goal of improving professional development [10–12]. ‘Reflection-on-action’ involves revisiting an encounter to explore lessons learned, and how the experience can inform new learning[10]. Collectively, these activities are best achieved through longitudinal relationships with faculty who assimilate various sources of information (e.g., direct observation, multisource feedback) and provide customized appraisal and feedback, facilitating an exploration to identify improvement areas [13–20]. In recent years, the coaching concept has increased in medical education, seeking to move away from assessment and towards a learner’s efficient and effective professional growth and performance[21]. However, graduate medical education (GME) programs struggle to operationalize these activities, and frequently lack sufficient infrastructure to support longitudinal relationships between trainees and faculty [13,22,23]. Trainees often interact with supervising physicians, in short, fragmented rotations, limiting opportunities for meaningful coaching and trust-building[24]. Although investigations into new education models are required to achieve CBME, these models are not well described [22,25–27].

In 2013, our Department of Medicine and Internal Medicine (IM) Residency Program implemented a coaching program in which residents were paired with a faculty coach for the duration of their training to enhance their professional development. The objectives of this manuscript are to: (1) describe the process and strategies for developing and implementing a longitudinal coaching program that evolved in response to challenges, (2) characterize residents’ professional learning plans (PLPs) and their alignment with IM EPAs, and, (3) examine key lessons learned to guide other residency programs in the development of coaching programs.

## Methods

### Setting

In 2012, the IM Residency Program at Penn State College of Medicine identified coaching for residents, specifically in achieving entrustment in EPAs, as an improvement area [28,29]. Led by the Vice Chair for Education (D.R.W.), a planning committee designed the Jeffries Educational Mentors and Scholars Program (JEMS, named after a senior faculty member, Dr. Graham Jeffries).

### Coaching program

#### Goals and recruitment

The program’s goal was to enhance residents’ achievement of competence through direct observations by coaches, facilitated reflection-on-action related to disorienting dilemmas, and targeted professional learning plans (PLPs) [16,19,28–30]. At the outset, to avoid confusion amongst residents and faculty, we used the term ‘mentor’ but purposefully used a coaching paradigm. We specifically sought to help residents to effectively and efficiently develop skills that aligned with the IM EPAs. Additionally, we sought to develop life-long skills of self-monitoring and self-directed learning [21,31,32]. We allowed resident–coach interactions to evolve over time, and focused on effective problem-solving, developing trust, and residents’ self-identified strengths and improvement areas [30,33,34]. Coaches were competitively selected through an application process. The application required potential coaches to provide a personal statement indicating their motivations for being part of the program. The applicant’s approach and developmental perspective was weighed heavily in the selection process. Selected coaches were all IM trained and practicing in an IM-based specialty, and provided 5% effort support per year. Faculty members’ education related to the coaching program occurred through regular, ongoing meetings with leadership (D.R.W., J.S.M-H), which included focused faculty development.

#### Process of coaching program

All categorical IM residents (n = 60) were matched with one faculty member (n = 20) who coached 3–4 residents across all postgraduate years (PGY1-3). In the first year, only PGY1-2 residents were assigned a coach. In subsequent years, assignments were maintained, and incoming PGY1 residents were paired with a coach during orientation. Programmatic modifications and improvements were informed by monthly, one-hour coach meetings, with discussions focused on challenges and successes. Additionally, strategies to improve and foster self-directed learning were reviewed, as well as mentoring and coaching skills training. Notes were recorded by program leadership (D.R.W., J.S.M-H). Based upon this process and subsequent changes made in the program over the first three years, we have articulated steps, objectives, a suggested approach, challenges, and strategies for a resident–coach program (Table 1).

**Table 1.** Steps, objectives, approach, challenges, and strategies for implementing a coaching program for internal medicine residents.

Steps	Objectives	Approach	Potential Challenges	Strategies
Preparation for resident–coach meeting	Prompt resident reflection on prior PLPs and progress Identify disorienting dilemmas for subsequent reflection and discussion	Introduce goals of coaching program to residents by residency and coaching program leadership Contact resident and schedule meeting Reminder about the need for identifying disorienting dilemmas or ‘what went well’ Consider sending notes from a prior meeting Coach observes resident in clinical settings to assist in identifying disorienting dilemmas	Initial trust-building and mutual conceptualization of goals may not be met May find difficulty in finding mutually conducive times between resident and coach to meet Coach observations may be seen as obtrusive and artificial, limiting value Maybe lack of accountability of who is responsible for scheduling and tracking meetings (coach or resident)	Hold orientation session with coaches and residents during the first week to describe goals/expectations Encourage/require 3-year mentor commitment to maximize continuity and rapport with residents Allow for fluid meeting methods (e.g., phone calls) Focus on residents self-reflection and identification of disorienting dilemmas or ‘what went well’ Identify time in residency schedule for meetings (i.e., during ambulatory block every 6–8 weeks)
During resident–coach meeting	Engage in reflective practice on challenges in clinical care Reflect upon emerging challenges in professional development Develop new professional learning plans	Perform ‘check-in’ with resident Update; schedule/rotations; health/wellness Review past meeting discussions and PLPs Focus of discussions: ‘Disorienting dilemmas’ and/or ‘best/worst days’ Clinical challenge Work–life balance Career planning; networking support Scholarship Patient connections – humanism in practice What went well recently; methods to sustain Review plans and next steps Next meeting date; agenda for next meeting	Residents may struggle to identify disorienting dilemma or area for improvement Resident PLPs may be superficial or lack depth Meetings may be viewed as ‘too prescriptive’ and ‘problem focused’	Use prompts and provide examples of potential areas of struggle to aide residents in finding challenges Facilitate honest discussion about weaknesses Focus meetings on ‘goal setting’ in addition to ‘disorienting dilemmas’ Use ISMART (important, specific, measurable, accountable, realistic, timeline) goals for meetings. Assign residents at same level of training to coach to facilitate exchange of common issues With permission, review recent clinic evaluations to promote self-reflection and goal-setting
Follow up after resident–coach meeting	Promote ongoing reflection-on-action, and PLPs	Review/critique resident’s summary via email Initiate periodic contact for updates on progress Colleague faculty/CCC may reach out to coaches to inform resident performance	Resident may dislike requirement to complete email summary Resident may be concerned about coach’s relationship with IM program, CCC, and reviewing evaluations Relationship may seem ‘forced’ if resident goals differ from coach specialty Resident may view meetings as onerous if they are seen as too frequent	Articulate and remind residents of coaching role Clearly articulate different roles between coach/CCC Focus discussions and expectations on coach facilitation of resident’s career needs Coach serves as ‘matchmaker,’ helping resident find career mentor, especially among other coaches Coach/resident mutually agree on realistic time frame for next meeting
Coach meetings	Review meetings, challenges/successes Perform structured review and targeted adjustments to program plan	Open discussion of commonalities and unique issues from mentee meetings and follow-up Develop a ‘toolkit’ of tactics for maximizing the effectiveness of resident–coach meetings Identify/discuss challenges, and plan a unified approach to address them from coaching group	Finding a time to involve all coaches versus multiple smaller meetings which limits the exchange of ideas	Optimize meeting time and provide alternative methods of attendance (web-based, conference calls) Use other experts to provide coach training; offer CME for some mentor meeting events

CCC = Clinical Competency Committee; CME = Continuing Medical Education; PLPs = professional learning plans

Each resident–coach pair was expected to meet routinely throughout the year, with the initial goal of meetings approximately every six to eight weeks. Prior to meetings, residents were asked to reflect on their clinical rotations and self-identify improvement areas. ‘Disorienting dilemmas’ and ‘best/worst’ days were used to stimulate this process (Appendix 1). Initially described by Mezirow, the disorienting dilemma includes clinical experiences prompting uncertainty or discomfort and pinpoints learning opportunities [35,36]. We used this reflective practice model as a starting point for resident–coach meetings[37]. ‘Worst days’ provided targets for resident–coach discussions to discover underlying disorienting dilemmas. In contrast, ‘best days’ provided positive reinforcement that could identify areas of strength around which to build PLPs for other issues. The expected outcome of each meeting was a PLP that was focused on an EPA (if applicable), or other area of learning need as articulated by the resident. In the program’s second and third years, residents were expected to email a summary of the resident–coach discussion, including PLPs, to their faculty coach, followed by coaches responding with comments as needed. These summaries were copied to a secure file in the program office.

#### Program evaluation and data analysis

The first aim of this program evaluation was to articulate the process and strategies for developing and implementing a coaching program that could be used by other residency programs. Based upon the process described above, including the survey administered after year three, coaches’ suggestions made during monthly faculty development programming, and several informal discussions between coaching program leadership and IM residency leadership, we made significant programmatic changes over the first three years. We have collaborated and agreed upon the steps, objectives, suggested approach, challenges, and strategies for the resident–coach program that are described in this manuscript.

The second aim of this manuscript was the characterization of disorienting dilemmas and PLPs, and their alignment with IM EPAs. We explicitly chose to examine EPAs because our program’s goal at outset was to determine whether our longitudinal mentor-coach model was effective in assisting residents in the development of entrustment. However, anticipating that other meaningful data were addressed in resident–coach interactions and, as suggested by interpretive qualitative research, we also sought to find additional key themes within the transcripts[38]. Thus, a hybrid qualitative analysis was performed – deductive thematic analysis with respect to the pre-specified EPAs and an inductive, exploratory examination of emergent themes not pre-specified in the EPAs. Two investigators (J.S.M-H., K.L.K.) independently analyzed all email summaries [38,39]. Each investigator independently coded half of data. In this first half of coding, the two investigators explicitly examined the transcripts for discussions that addressed the EPAs. In addition, the investigators also identified key emergent themes that were not explicitly articulated in the EPAs. After coding half the data, the two investigators came together to jointly identify and agree on additional themes, as well as refine their coding scheme for the EPAs. The two investigators then independently coded the remaining data, met again to resolve all coding discrepancies through discussion and arbitration. Finally, to determine which EPAs and additional themes were most commonly discussed, we examined frequencies. Representative examples of qualitative data constructs are provided.

The third aim was to characterize the key lessons learned, which was inclusive of barriers and facilitators to coach program development. To better inform these lessons, three years after implementation (May 2016), we sent an anonymous electronic survey to residents and coaches with open-ended prompts soliciting perceived challenges and potential improvements (Appendix 2). Survey responses were analyzed by two investigators (J.S.M-H., D.R.W.), and all investigators agreed upon results. The Institutional Review Board deemed the project as quality improvement and exempt from review.

## Results

### Program process and strategies

Table 1 articulates the steps, program objectives, our approach to meeting the objectives, challenges we encountered, and strategies used to overcome those challenges. This table also incorporates several barriers and challenges to program implementation as described below in Lessons Learned. Key steps to program development include adequate preparation for resident–coach meetings, creating meeting content that is relevant and meaningful for the resident, developing strategies for appropriate follow-up after the meeting, and ongoing professional development for faculty coaches to ensure adherence to best practices for fostering a developmental approach for residents.

### Disorientating dilemmas, professional learning plans, EPAs

To characterize the disorienting dilemmas and PLPs, we initially examined data from submitted meeting summaries, which included a total of 119 resident–coach meeting summaries. Sixty-five unique residents (81%) submitted at least one summary containing a disorienting dilemma and/or PLP during our review. The frequencies by year included: PGY1 = 51 meeting summaries (160 coding references); PGY2 = 48 summaries (128 coding references); PGY3 = 20 summaries (35 coding references). We identified a total of 323 disorienting dilemmas with PLPs mapped across all 16 EPAs, and four additional themes: (1) work–life balance, (2) career planning, (3) teaching skills, and, (4) research/scholarship [28,29]. Table 2 depicts the mapping and frequency of disorienting dilemmas/PLPs to EPAs and additional themes from data submitted during the second and third years of the program.

**Table 2.** Frequency and representative examples of disorienting dilemmas and professional learning plans mapping to internal medicine entrustable professional activities and identified discussion themes.^a.^

Entrustable Professional Activities	PGY1 n (%) of 108	PGY2 n (%) of 90	PGY3 n (%) of 21	Disorienting Dilemmas	Professional Learning Plans
EPA1: Manage care of patients with acute common diseases across multiple care settings	5 (5)	3 (3)	1 (5)	PGY1-challenge with keeping up with note writing during an internal medicine service, often times submitting notes in evening after shifts	Implements new method of writing portions of note prior to rounds, supplementing updated information following rounds and during work tasks.
EPA2: Manage care of patients with acute complex diseases across multiple care settings	7 (7)	5 (6)	-	PGY2-uncomfortable with managing complex patients during short clinic visits	Identifies ‘must-not-miss’ elements (max of 2) in the care of each complex patient, with plans to revisit additional concerns at subsequent visit.
EPA3: Manage care of patients with chronic diseases across multiple care settings	6 (6)	3 (3)	-	PGY1-poor understanding of services provided at skilled nursing facility and rehabilitation hospital following patient’s discharge	Requests elective in rehabilitation unit; articulates differences/similarities acute vs. rehabilitation setting to coach at follow up meeting.
EPA4: Provide age-appropriate screening and preventative care	-	2 (2)	-	PGY2-frustrated with a patient who consistently did not follow recommendations regarding healthy habits (e.g., diet)	Avoids ‘paternalistic’ approach in encounters, focusing on patient-identified barriers to healthy lifestyle (motivational interview techniques).
EPA5: Resuscitate, stabilize, and care for unstable or critically ill patients	8 (7)	1 (1)	-	PGY1-apprehensive about responsibility for code calls as s/he assumes new PGY2 role.	Attends as many codes as possible at end of PGY1 year; identifies effective strategies used by code leaders (i.e., how/when leaders ask for input)
EPA6:Provide perioperative assessment and care	-	3 (3)	-	PGY2-feeling unprepared to practice perioperative medicine.	Coach and resident review perioperative medicine algorithms and resources. Resident plans consult rotation during elective time.
EPA7: Provide general internal medicine consultation to nonmedical specialties	2 (2)	2 (2)	1 (5)	PGY1-discomfort receiving information from a consultant in an unfamiliar format.	Examines how non-medical specialties utilize different communication styles; consider how those receiving consults would utilize information.
EPA8: Manage transitions of care	9 (8)	7 (8)	-	PGY1-identified lack of appropriate follow-up and pass-off at discharge.	Identifies patients without PCP care, takes on PCP role or ensures follow-up and communication with a colleague who will be PCP.
EPA9: Facilitate family meetings.	3 (3)	3 (3)	-	PGY2-unprepared for family meetings in end-of-life care.	Reviews literature on family meetings, identifies aspects of family meeting s/he would like to improve, in subsequent rotation, practices one strategy.
EPA10: Lead and work within interprofessional health care teams.	18 (17)	14 (16)	2 (10)	PGY2-identified inadequate communication between medicine team and nurses, potentially compromising care.	Implements plan to identify each patient’s nurse and ensure nursing presence during rounds.
EPA11: Facilitate the learning of patients, families, and members of the interdiscplinary team.	7 (7)	2 (2)	-	PGY1-concerned about patient expressing dissatisfaction with care; resident struggled communicating with patient without adversely affect patient-doctor relationship.	Reviews medical ethics literature and developed plan to show empathy and respect for the patient while maintaining professional standards.
EPA12: Enhance patient safety.	6 (6)	4 (4)	-	PGY2-concerned about antimicrobial stewardship skills.	Sets time-course for antibiotics/daily reminders to re-evaluate; coach reminds resident to consider applying to all aspects of plan.
EPA13: Improve the quality of health care at both the individual and systems level.	13 (12)	15 (17)	4 (19)	PGY2-identified goal to improve cost-conscious care, but realizes that s/he cannot personally identify cost of labs/imaging studies, etc.	Increased contact with ancillary professionals who can serve as experts including pharmacists, care coordinators and case managers.
EPA14: Advocate for individual patients.	1 (1)	2 (2)	-	PGY2- uncertainty with addressing concern about suboptimal management with senior team members.	Collects objective data (e.g., vital signs, literature citations) to share with care team, while maintaining kind and cooperative demeanor.
EPA15: Demonstrate personal habits of lifelong learning.	13 (12)	19 (21)	11 (52)	PGY3-anxious about upcoming board exam	Examines areas of weakness identified on in-training exams; complete 5–10 MKSAP questions each weekday in these areas.
EPA16: Demonstrate professional behavior.	10 (9)	5 (6)	2 (10)	PGY2-concerned about whether or not to attend memorial service for a deceased patient.	Reads pertinent manuscripts on topic, reflects on encounter, writes a reflection and discusses with coach.
**Additional Themes**	**PGY1 n (%) of 52**	**PGY2 n (%) of 38**	**PGY3 n (%) of 14**	**Disorienting Dilemmas**	**Professional Learning Plans**
Theme1: Work–Life Balance	17 (33)	3 (8)	2 (14)	PGY1-worried about anticipated increase in clinical demands and potential to adversely affect time with partner.	Plans for ‘expected minimums’ (e.g., one sit-down dinner together per week, go to bed at the same time 3x per week).
Theme 2: Career Planning	16 (31)	15 (40)	8 (57)	PGY2-uncertainty about career choice – expressed interest in specialty, but was unsure whether it was a good match.	Considers rotations that will enhance skill development/knowledge of specialty; meets with colleagues at outside institutions in specialty.
Theme 3: Teaching Skills	4 (8)	6 (16)	1 (7)	PGY2-frustrated with teaching medical students of diverse personality types (e.g. quiet, over-zealous, disinterested)	Sets expectation of self-directed learning at beginning of rotation; each learner will self-identify at least one objective for the rotation.
Theme 4: Research and scholarship	15 (29)	14 (37)	3 (21)	PGY1-struggling with identifying network of potential mentors for research projects.	Coach identifies two potential research mentors; gives resident further steps to inquire about other potential mentors

^a^ A total of 119 transcripts were analyzed from 65 residents (possible 80 unique residents over 2 years of program). Frequencies include: PGY1 = 51 summaries, 160 different code references; PGY2 = 48 summaries, 128 different code references; PGY3 = 20 summaries, 35 different code references. Not all resident–coach teams met at prescribed intervals and not all meetings included documented summaries. Shading of cells indicate the density of percentage: 0 (white), 1–5 (lightest gray), 6–10 (light gray), 11–20 (gray), 21–30 (dark gray), ≥30 (darkest gray)

Resident–coach discussions were dynamic and changed throughout the course of residents’ training. During PGY1, most-commonly discussed dilemmas/PLPs aligned with leading and working within interprofessional health-care teams (EPA10), research/scholarship (Theme 4), career planning (Theme 2), and work–life balance (Theme 1). During PGY2, most-commonly discussed dilemmas/PLPs aligned with improving quality of care (EPA13), demonstrating personal habits of lifelong learning (EPA15), research/scholarship (Theme 4), and career planning (Theme 2). During PGY3, most-commonly discussed dilemmas/PLPs aligned with demonstrating professional habits of lifelong learning (EPA15) and career planning (Theme 2). Later in residency, resident–coach discussions shifted away from EPAs towards more traditional mentoring topics, including research, fellowship applications, and professional transition into fellowship/practice. Table 2 shows representative examples of disorientating dilemmas and PLPs addressed in the coaching program.

### Key lessons learned

Key lessons learned are derived from surveys completed at the end of year three of the program (60 residents and 20 faculty invited, with 14 (24%) and 12 (60%) completing the survey, respectively) and outputs of coach meetings. Three higher-level key lessons learned in the design and implementation of our resident coaching program in an IM residency program were identified by investigators.

### Coordination and authenticity of direct observations were challenging

Our initial plan was to ground relationships in observations performed by coaches, allowing for feedback and subsequent reflection-on-practice[30]. However, this model met significant challenges in the early months. Coaches struggled to find mutually available times for observation, and as a result, infrequently observed residents. Factors limiting these observations included 1) resident perceptions that observations hindered workflow, 2) resident concern that coaches were summative ‘evaluators’ rather than facilitators of formative feedback, and 3) resident tendency to select low-complexity patients, limiting educational benefits. After six months, we were pressed to modify the program to focus less on direct observation and more on alternative sources of information, such as residents’ self-assessment, rotation evaluations, and facilitated reflection and goal-setting[20]. This change was consistent with programmatic goals to avoid confounding the coach’s role with ‘evaluator,’ and maintaining an environment conducive to trust-building between resident and coach[16]. Moreover, the four additional themes we identified strongly suggest that residents desired their coaches work with them on areas not traditionally assessed by evaluators. However, this change limited the coach’s opportunities to assess whether a learner could be entrusted, thereby diminishing one of the key goals of the program.

### Residents and coaches had challenges in identifying disorienting dilemmas

Residents were occasionally frustrated by the request to identify disorienting dilemmas when they perceived none had occurred. This also became a challenge for coaches, as some meetings were less productive when no challenge was identified and discussed. This prompted several modifications, including encouraging residents to proactively monitor for challenges in real-time, focusing meetings on ”best/worst” days, allowing resident–coach pairs more flexibility in follow-up time intervals, and using the ISMART (important-specific-measurable-accountable-realistic-timeline) mnemonic to establish learning objectives[40]. This allowed for less-scripted, open-ended dialogue between resident and coach, challenged residents to identify PLPs, and created greater accountability for follow-up with the coach.

Residents’ ability to self-assess and identify disorienting dilemmas has been and continues to be a significant challenge. Compared to ‘time-in-seat’ education, CBME requires collaboration between resident and coach, with responsibility on the learner to be active in reflecting, assessing, and developing PLPs[9]. Residents, therefore, must possess self-directed learning, self-reflection, and self-assessment skills, which is lacking in some trainees [9,41,42]. In our experience, residents variably possess these skills, and this was manifest in residents’ struggles to identify disorienting dilemmas. Closing this gap could include integrating real-time faculty assessment into clinical processes, allowing coaching to more appropriately demonstrate reflection-on-action [16,30,43]. However, we designed this program based on the perceived lack of ‘coaching’ by ward attendings and clinic preceptors. Future work could explore the gap between non-observing coaches and faculty preceptors who do have opportunities to observe, and determining how to use both in summative entrustment decisions.

### A shared mental model for coaching role must exist amongst all stakeholders

At the program’s start, residents, coaches, and Clinical Competency Committee (CCC) identified the need to differentiate stakeholder roles, as some viewed the coach as ‘program police,’ seeking to identify struggling residents. Additionally, residents and coaches often had different expectations of the program. We needed to articulate the relationship between the program and CCC, and coach’s role to all stakeholders [44,45]. Delineation of clear expectations allowed high-yield interaction between CCC and coaching program. More importantly, proactive transparency by the program and coaches about the relationship with CCC preserved a safe environment for honest discussions. The relationship with program leadership has become increasingly beneficial in both directions, with opportunities for resident advocacy by coaches, and coaching interventions based on CCC feedback to resident–coach pairs.

## Discussion

In response to our need to enhance longitudinal relationships between residents and faculty, we implemented a coaching program that supports residents’ professional development. This program was modified over time based on challenges and successes articulated by the residents and coaches. The transition from less granular, ‘time-based’ assessments to Competency-Based Medical Education (CBME) and entrustment requires more direct workplace-based observations of trainees, provision of high-quality feedback, and use of highly reliable assessment tools, all of which require transformation in processes, resources, and faculty expectations [13,46–48]. Our program was designed upon a deliberate-practice approach using reflection-on-action to drive identification of disorienting dilemmas, and aligned professional learning plans facilitated by faculty coaches [6,8,11,17,18,22,27,30,37,49]. This approach has great potential for advancing the goals of the Next Accreditation System, CBME, and residents’ professional development. Moreover, a flexible approach allows residents to prioritize their own problem-solving, and to articulate additional needs as they arise.

Although we identified several successes and benefits to the coaching program, our work highlights several challenges in how faculty coaches could be used in entrustment decision-making. Despite significant investment, we struggled to pragmatically ensure all three components of entrustment – direct observation, feedback, and reflective practice – were fully integrated into the coaching program, with direct observation posing a unique logistic challenge [3,50]. Ideally, direct observation would be performed by supervisors in clinical settings, but as has been previously identified, residents’ interactions with faculty supervisors are often fragmented in rotations, limiting sustained working relationships that allow for customized improvement of clinical skills [14,15]. The focus of resident–coach discussions often tended away from coaching towards traditional mentoring activities, including career planning and wellness. This shift away from EPAs may have been due to lack of direct observation opportunities, a culture less accustomed to coaching behaviors, and/or a previously undiscovered need for residents to have an outlet for non-threatening conversations about work–life balance, wellness, and career development. Nevertheless, it is important to note that our focus on EPAs in the resident–coach interaction likely increased residents’ attention to these learning areas. Even lacking direct observation, we suspect this focus on EPAs brought these competencies to the forefront of residents’ minds, which is reflected in the frequencies of EPAs discussed in resident–coach meetings. Thus, the coaching model shows promise as one aspect of a multi-pronged program designed to examine these competencies.

Regardless, there is a pressing need to transform clinical learning environments at the systems level to allow for longitudinal faculty coaching to meet these needs[51]. This requires significant changes, such as designing GME programs that link residents with faculty over longer time periods, faculty development in coaching skills, reconsidering clinical faculty roles, and examining the balance between clinical service and education requirements [13,46,52]. With clinical productivity pressures, faculty are increasingly being pushed to meet revenue benchmarks, which can be at odds with authentic coaching of residents in CBME frameworks.

Several limitations must be considered. Most notably, the number of submitted resident–coach meeting summaries was low, potentially decreasing the reliability of these findings. Theoretically, based on the suggested frequency of resident–coach meetings (approximately seven times/year) and number of residents in our program, we conservatively estimate our data accounts for at least 14% of the resident–coach interactions that occurred during the study. However, informal feedback from the coaches suggests that our data capture a larger proportion of the meetings that took place. Meetings were challenging to schedule (thus occurring less frequently than the ‘ideal’ of approximately seven/year). Our data collection was also challenged by the fact that, even after productive resident–coach interactions, residents did not always initiate meeting summaries as requested. An additional limitation to consider is that the single-center implementation suggests modifications would be required for use in other settings. We had 5% departmental FTE support for coaches, which is a substantial input of resources that may not be replicable at other institutions. Lastly, we describe implementation features and do not report the impact on residents’ behaviors or practice. Further study is needed to determine whether this coaching model can lead to changes in the systems or practice environment and whether these changes can be sustained.

In conclusion, we designed and implemented a longitudinal coaching program for Internal Medicine residents with the goal of fostering resident abilities in reflective practice to better achieve competency. Future work will need to explore strategies that blend longitudinal resident–coach clinical relationships with coaching processes to allow for residents’ professional development. Future work should also link this coaching work with resident learning outcomes, such as improvements in resident reflection-on-action skills. Ultimately, the strength of any coaching program should be evaluated in the context of how graduating residents practice and how they provide coaching for the next generation of learners.

## Data Availability

This report data is available through the corresponded author.

## Appendix Appendix 1. Instructions for Residents Prior to JEMS Faculty Mentor Meetings

**Table:**

“Now that you have your schedule, please contact your JEMS faculty now or in the next week or two to set up a time to meet during this upcoming block. Do not wait until the block starts – that approach often makes it hard to find a time that works for both of you.
In preparing for the meeting, we would also like you to think about something you would like to work on with your JEMS mentor: A specific area that you would like to improve on Some situation you encountered during the course of your work that made you uncomfortable, made you feel like you needed help or otherwise challenged you in some way – a ‘disorienting dilemma’. Your ‘worst day’ or ‘best day’ – and why.
Think about this before your meeting and be prepared to come up with a learning plan to address it. We prefer that: It is not strictly content or procedural skill related It relates directly to your experience and your needs. We feel that most ‘disorienting dilemmas’ will map to one of the ‘Entrustable Professional Activities’ (EPAs) listed on the attached document – it would be helpful if you could look these over as well.

## Appendix Appendix 2. Survey Items Sent to Internal Medicine Residents and Coaches in 2016

**Table:**

1. What is your understanding of the goals of JEMS?
2. What is the most effective aspect of JEMS for you?
3. What would you change to improve the quality of JEMS?
4. Please add any additional comments about JEMS.
